# Trends in vasectomy and sexually transmitted diseases in Chile: findings from robust national databases

**DOI:** 10.1590/0102-311XEN129323

**Published:** 2024-03-11

**Authors:** Daniela Toledo, Cinthya Urquidi, Alejandro Sepúlveda-Peñaloza, Rodrigo Leyton

**Affiliations:** 1 Facultad de Medicina, Universidad de los Andes, Santiago, Chile.; 2 Universidad de los Andes, Santiago, Chile.; 3 Hospital Clínico “Gral. Dr. Raúl Yazigi J.”, Fuerza Aérea de Chile, Las Condes, Chile.

**Keywords:** Vasectomy, HIV, Sexually Transmitted Diseases, Vasectomía, VIH, Enfermedades de Transmisión Sexual, Vasectomia, HIV, Infecções Sexualmente Transmissíveis

## Abstract

A controversy about the increase or decline of vasectomy is emerging; however, the evidence is still scarce in Latin America. This ecological study analyzed the vasectomy and sexual transmitted diseases (STD) trends over a period of 10 years in Chile and determined if there is any relationship between them. We conducted a mixed ecological study using secondary and representative data on the number of vasectomies and STD cases from 2008 to 2017. Vasectomy rates were calculated for age-specific groups of men aged 20-59 years, and specific STD (HIV, chlamydia, gonorrhea, trichomoniasis, and syphilis) for the same period. Multivariate negative binomial regression models were fitted to evaluate rate trends and relationships. The mean vasectomy age was 40.3 years, with no significant differences between the years of the study (p = 0.058). The overall vasectomy rate significantly increased from 2008 to 2017 (p < 0.001), with differences between age groups (p < 0.001). The most significant increase was observed in men aged 30-49 (p < 0.001). The STD rates significantly increased (p < 0.05) during the study period. A significant positive correlation was found between vasectomy and gonorrhea incidence rates (p = 0.008) and an inverse correlation was found with hepatitis B incidence rates (p = 0.002). Vasectomy trends and STD rates significantly increased from 2018 to 2017 in Chile. especially among men aged 30-49 years. The relationship between vasectomy and STD increments suggests a new risk factor for reproductive and sexual health policies to aid controlling the HIV and STD epidemic.

## Resumen

Se viene surgiendo una controversia sobre el aumento o la disminución de la vasectomía; sin embargo, la evidencia aún es escasa en América Latina. Este estudio ecológico analizó las tendencias de la vasectomía y las enfermedades de transmisión sexual (ETS) durante un período de 10 años en Chile y determinó si existe alguna relación entre ellas. Este es un estudio ecológico mixto que utiliza datos secundarios representativos sobre el número de vasectomías y casos de ETS de 2008 a 2017. Las tasas de vasectomía se calcularon por grupos de edad teniendo en cuenta a hombres entre 20-59 años de edad y ETS específicas (VIH, clamidia, gonorrea, tricomoniasis y sífilis) para el mismo período. Los modelos de regresión binomial negativa multivariante se ajustaron para evaluar las tendencias y las relaciones entre las tasas. La edad media de la vasectomía fue de 40,3 años, sin diferencias significativas entre el período del estudio (p = 0,058). La tasa general de vasectomía tuvo un incremento significativo para el período de 2008 a 2017 (p < 0,001), con diferencias entre los grupos de edad (p < 0,001). El incremento más significativo se observó en hombres de entre 30-49 años (p < 0,001). Las tasas de ETS tuvieron una alza significativa (p < 0,05) durante el período de estudio. Hubo una correlación positiva significativa entre las tasas de vasectomía y las tasas de incidencia de gonorrea (p = 0,008) y una correlación inversa con las tasas de incidencia de hepatitis B (p = 0,002). Se concluye que las tendencias de la vasectomía y las tasas de ETS presentaron un incremento significativo en período de 2008 a 2017 en Chile, especialmente entre los hombres de entre 30-49 años. La relación entre la vasectomía y el incremento de las ETS puede ser un nuevo factor de riesgo para las políticas de salud sexual y reproductiva que puede ayudar a controlar la epidemia del VIH y las ETS.

## Resumo

Uma controvérsia sobre o aumento ou declínio da vasectomia está surgindo; no entanto, as evidências ainda são escassas na América Latina. Este estudo ecológico analisou as tendências de vasectomia e doenças sexualmente transmissíveis (DST) em um período de dez anos no Chile e determinou se há alguma relação entre elas. Este é um estudo ecológico misto utilizando dados secundários e representativos sobre o número de vasectomias e casos de DSTs de 2008 a 2017. As taxas de vasectomia foram calculadas por grupos etários, considerando homens de 20-59 anos de idade e DSTs específicas (HIV, clamídia, gonorreia, tricomoníase e sífilis) para o mesmo período. Modelos de regressão binomial negativa multivariada foram ajustados para avaliar tendências e relações entre taxas. A média de idade da vasectomia foi de 40,3 anos, sem diferenças significativas entre os anos do estudo (p = 0,058). A taxa geral de vasectomia aumentou significativamente de 2008 a 2017 (p < 0,001), com diferenças entre grupos etários (p < 0,001). O aumento mais significativo foi observado em homens de 30-49 anos (p < 0,001). As taxas de DST aumentaram significativamente (p < 0,05) durante o período de estudo. Houve correlação positiva significativa entre as taxas de vasectomia e as taxas de incidência de gonorreia (p = 0,008) e uma correlação inversa com as taxas de incidência de hepatite B (p = 0,002). As tendências de vasectomia e as taxas de DST aumentaram significativamente de 2008 para 2017 no Chile, especialmente entre homens de 30-49 anos. A relação entre vasectomia e incrementos de DST sugere um novo fator de risco para políticas de saúde sexual e reprodutiva para auxiliar no controle da epidemia de HIV e DST.

## Introduction

The 2030 Agenda for Sustainable Development includes sexual and reproductive health and rights, including contraception and sexually transmitted diseases (STD) ^1^. For family planning and STD prevention, the World Health Organization (WHO) recommends community behavioral interventions and individual contraceptives. For men, vasectomy and condoms are the only available methods of contraception. Vasectomy is a simple surgical procedure with a low complication rate; however, despite its higher effectiveness for contraception, it is a non-barrier method and cannot prevent STD, like condoms ^2^.

A controversy about the increase or decline in the number of vasectomy cases is emerging; however, the evidence is still scarce and is not representative of the context of access to the health systems of different countries. For example, in the United States, a representative national survey reported a decline in vasectomy rate from 2002 to 2017 ^3^. Meanwhile, in New Zealand, Canada, and the United Kingdom, the cross-sectional prevalence rates ranged from 20% to 25% ^4^, with no available reports in Latin America. Thus, epidemiological studies are needed to assess the trends in different settings.

According to the WHO, chlamydia, gonorrhea, trichomoniasis, and syphilis remain the most prevalent preventable STD worldwide, affecting 376.4 million people aged 15-49 years ^5^. Similarly, the HIV accounted for 1.94 million new infections in 2017, with differences between countries in the incidence and mortality rates ^6^. The HIV mortality rates in countries in Asia and Africa are predicted to decline by 2030. Nevertheless, the trends in HIV incidence and mortality show little progress in Latin America ^6^, including Chile ^7^.

Recently, two small studies have suggested that increasing the number of non-barrier contraceptive methods could influence sexual behaviors, subsequently increasing the risk of STD due to perceived lower pregnancy rates ^8^. After the approval of the “over-the-counter” policy for selling emergency contraceptive pills in the United States, the incidence of STD among women increased by 13.4% ^9^. Likewise, a retrospective secondary analysis of 51,000 healthcare professionals revealed a higher vasectomy rate among men with a history of STD ^10^. Addressing this hypothesis from an ecological point of view would provide evidence for the relevance of planning robust studies at the individual level.

Therefore, the primary objective of this study was to analyze vasectomy trends over a 10-year period in Chile, leveraging a robust and nationally representative dataset. The secondary objectives were to describe STD trends in the same period and determine whether a relationship exists between these vasectomy trends.

## Methods

### Study design and data sources

We conducted a mixed-ecological study using secondary and representative data on the number of vasectomies and STD cases from 2008 to 2017. For rate calculations, we used data from national epidemiological reports and the projected population-based on the 2017 census from the Chilean Institute of Statistics (INE, acronym in Spanish). We selected this period since the data on HIV and STD at the time of the analysis in Chile were only available till 2017, and we set the period 10 years back to make it comparable with the series of another study ^11^. Moreover, this study design allowed us to examine relationships between vasectomy changes over time and specific STD trends by multivariate regression models analysis.

The Department of Health Statistics and Information from the Chilean Ministry of Health (DEIS, acronym in Spanish) manages datasets on vasectomies, which are publicly and anonymously available for free (https://deis.minsal.cl). We collected a number of HIV and STD cases from free-access epidemiological reports ^12^. Consequently, this study did not require ethical approval, participation consent, or administrative permission. Our analysis was performed in accordance with the *Declaration of Helsinki* and Chilean regulations.

### Vasectomy

We extracted the number of vasectomies performed annually and at the individual level from the hospital discharge statistical reports provided by the DEIS. The DEIS registry included the following variables: date of hospital discharge, healthcare insurance, age, sex, length of stay, hospital discharge diagnosis according to the 10th revision of the International Classification of Diseases (ICD-10), and local hospital discharge codification assigned by the Chilean public healthcare system. We included data from men aged 20-59 years since men are most reproductive during this age range according to the INE criteria. We used the hospital discharge diagnosis of “bilateral vasectomy”, which corresponds to local codification n. 1902076. We did not use the ICD-10 code since it is not widely used by urologists in Chile. Nevertheless, the local codification has good reliability and systematic registration. The date of vasectomy, healthcare insurance, and age (categorized into four groups: 20-29, 30-39, 40-49, and 50-59 years) were also analyzed.

The overall annual vasectomy rates were calculated as the ratio between the number of vasectomies reported in year *t* and the projected male population aged 20-59 years in the same year *t* per 100,000 habitants, where *t* is each year of the study (*t*: 2008, 2009, ..., 2017). These rates were also calculated for each age group.

### Sexually transmitted disease

We collected data on STD from the *Epidemiological Situation of Sexually Transmitted Infections in Chile, 2017* report ^9^, which is based on information from the national epidemiological surveillance system in Chile. These reports included new cases and rates of annual notifications of HIV and specific STD. We included records of HIV, gonorrhea, syphilis, and hepatitis B cases. Annual specific STD rates were calculated as the ratio of the total number of cases in year *t* and the projected population aged 20-59 years in the same year *t* per 100,000 habitants.

### Statistical analysis

A descriptive analysis of the absolute and relative frequencies of vasectomy was conducted according to age group and healthcare insurance. The mean ages for each year were estimated and compared using analysis of variance.

Negative binomial regression models were preferred for vasectomy and STD trends due to data overdispersion. Three models were fitted as follows: “Model 1” for overall vasectomy trends, “Model 2” to test the linear change by age group, and “Model 3” to find differences in the rate of change between age groups. The equation for Model 3 is as follows:

$$ln\left(E\left[v\right.|\left.t,e\right]\right)=\beta_{ο}+\beta_{1}t+\beta_{2}e+\gamma t,e+ln\left(P_{t,e}\right)$$

Where (*E*[*v*|*t*,*e*]) is the expected value of the vasectomy rate for year *t* (*t* = 2008, 2009, ..., 2017) and age group *e*. The offset term ln(*P*_*t,e*_ ) is the male population aged 20-59 years at year *t*. Coefficient β_0_ is the model intercept; coefficient β_1_ estimates the overall vasectomy trend; β_2_ is the vasectomy trends by age group (reference: 50-59 years); and γ_i,j_ is the interaction term between year and age group to test if the trends have different slopes.

The model that estimates the relationship between specific STD rates (outcomes) and vasectomy trends (exposure) is as follows:

$$ln\left(E\left[S\right.|\left.t,v\right]\right)=\beta_{ο}+\beta_{1}t+\beta_{2v}+\gamma t,v+ln\left(P_{t}\right)$$

Where (*E*[*S*|*t*,*v*]) is the expected rate of specific STD conditional for each year *t* and vasectomy rate *v*. The β_1_ estimates the STD trends over the years, β_2_ is the correlation of the vasectomy rate and outcomes, and γ_i,j_ is an interaction term between the year and vasectomy rate that estimates the effect of the slope of increase in vasectomy rates on specific STD rate trends. We also conducted the same analysis stratified by age group.

All analyses were performed using RStudio version 4.1.0 (https://rstudio.com/) and Stata version 16 (https://www.stata.com). Statistical significance was set at a p-value of < 0.05.

## Results

We included 7,361 vasectomy records collected from January 2008 to December 2017. Table 1 shows the distributions of the vasectomies by age group and healthcare insurance. The mean (standard deviation - SD) age of the men was 40.3 (±0.08) years, with no significant differences between the years of the study (p = 0.058). Vasectomy was performed mainly under a private healthcare insurance, representing 75.4% (n = 5,548) of all vasectomies.

**Table 1:** Vasectomy data distribution in Chile from 2008 to 2017.

	2008	2009	2010	2011	2012	2013	2014	2015	2016	2017
Mean age [years] at vasectomy (SD) *	41.6 (6.9)	40.2 (6.8)	40.1 (7.2)	40.5 (6.6)	40.3 (6.8)	40.4 (6.8)	40.3 (6.4)	40.4 (6.9)	40.5 (6.4)	39.9 (6.5)
Number of vasectomies	196	339	335	421	537	679	822	1,079	1,198	1,755
By healthcare insurance (%)										
Public	29 (14.8)	30 (8.9)	39 (11.6)	50 (11.9)	62 (11.6)	62 (9.1)	65 (7.9)	72 (6.7)	79 (6.6)	112 (6.4)
Private	126 (64.3)	237 (69.9)	199 (59.4)	308 (73.2)	347 (64.6)	503 (74.1)	648 (78.8)	861 (79.8)	937 (78.2)	1,382 (78.8)
Military	38 (19.4)	0 (0.0)	0 (0.0)	49 (11.6)	105 (19.6)	85 (12.5)	43 (5.2)	71 (6.6)	111 (9.3)	157 (9.0)
No healthcare insurance	3 (1.5)	7 (2.1)	4 (1.2)	2 (0.5)	5 (0.9)	5 (0.7)	6 (0.7)	14 (1.3)	13 (1.1)	20 (1.1)
By age groups [years] (%)										
20-29	6 (3.1)	21 (6.2)	23 (6.9)	20 (4.8)	23 (4.3)	30 (4.4)	34 (4.1)	49 (4.5)	38 (3.2)	91 (5.2)
30-39	71 (36.2)	142 (42.7)	143 (42.7)	166 (39.4)	228 (42.5)	280 (41.2)	346 (42.1)	449 (41.6)	515 (40.1)	766 (43.7)
40-49	91 (46.3)	142 (39.7)	133 (39.7)	195 (46.3)	234 (43.6)	306 (45.1)	371 (45.1)	466 (43.2)	538 (44.9)	760 (43.3)
50-59	28 (14.3)	34 (10.0)	36 (10.8)	40 (9.5)	52 (9.7)	63 (9.3)	71 (8.6)	115 (10.7)	107 (8.9)	138 (7.9)

SD: standard deviation.

* ANOVA p-value for equal means = 0.058.

### Vasectomy trends

**Figure 1:**
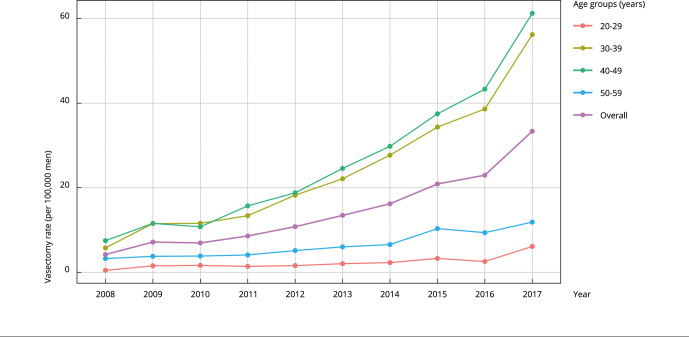
Vasectomy rate trends in men aged 20-59 years by age groups in Chile.

Figure 1 and Table 2 display the negative binomial regression model results for the vasectomy trends. The overall vasectomy rate significantly increased from 2008 to 2017 (Model 1: coefficient, 0.21; p < 0.001 in Table 2), from 4.3 per 100,000 men in 2008 to 33.6 per 100,000 men in 2017, representing a mean expected increase of 2.8 per 100,000 men per year. Meanwhile, the vasectomy rates were significantly different among age groups. The 20-29 years age group had a lower vasectomy rate than the reference group (50-59 years; Model 2: coefficient, -1.05; p < 0.001 in Table 2, Figure 1). By contrast, the 30-39 years and 40-49 years age groups showed higher vasectomy rates (Model 2: 1.30 and 1.38, respectively; p < 0.001 in Table 2, Figure 1). The interaction term between the time (years) and age group was statistically significant. The trend slope of the 30-39 years and 40-49 years age groups were positive and statistically different (Model 3: 0.06 and 0.07, respectively; p < 0.001 in Table 2, Figure 1) from the reference group (50-59 years).

**Table 2:** Negative binomial models for overall vasectomy trends in Chile from 2008 to 2017, and by age groups.

	Model 1 (β_0_: -10.16; AIC: 485.63)			Model 2 (β_0_: -10.97; AIC: 343.96)		Model 3 (β_0_: -10.61; AIC: 335.46)	
	Coefficient (SE)	p-value	Coefficient (SE)	p-value	Coefficient (SE)	p-value
Year *t*	0.21 (0.05)	< 0.001	0.21 (0.01)	< 0.001	0.16 (0.02)	< 0.001
Age group [years] *e* (reference: 50-59 e4)						
20-29 (e1)	-	-	-1.05 (0.08)	< 0.001	-1.32 (0.20)	< 0.001
30-39 (e2)	-	-	1.30 (0.06)	< 0.001	0.88 (0.13)	< 0.001
40-49 (e3)	-	-	1.38 (0.06)	< 0.001	0.94 (0.13)	< 0.001
Interaction *t* x *e* (reference: *t* x e4)						
*t* x *e*1	-	-	-	-	0.04 (0.03)	0.128
*t* x *e*2	-	-	-	-	0.06 (0.02)	< 0.001
*t* x *e*3	-	-	-	-	0.07 (0.02)	< 0.001

AIC: Akaike information criterion; β_0_: intercept of the model; SE: standard error of the coefficient.

Note: *t*: 2008, ..., 2017.

### Relationship between STD trends and vasectomy rates

Table 3 shows results of the negative binomial regression model, on the relationships between the specific STD trends and change in vasectomy rates. Model 1 results indicate that all specific STD rates significantly increased over the 10-year study period (p < 0.05). Model 2 results show a significantly positive relationship between vasectomy rates and gonorrhea incidence rates (0.021, p = 0.008). By contrast, a negative correlation with hepatitis B was found (-0.08, p = 0.002), which suggests that higher vasectomy rates were related to increased incidence rates of gonorrhea and lower incidence rates of hepatitis B. However, a relationship was absent between the vasectomy rates and the incidence rates of HIV and syphilis (Model 2, Table 3). The effects of change in vasectomy rate over time (interaction terms) on the trends of the STD incidence rates were estimated using Model 3. Over the years, the overall changes in vasectomy rate failed to show correlation with the specific STD rates.

**Table 3:** Negative binomial for sexually transmitted diseases (STD) trends in Chile from 2008 to 2017, and its relationship with vasectomy rates.

	Model 1		Model 2		Model 3	
	Coefficient (SE)	p-value	Coefficient (SE)	p-value	Coefficient (SE)	p-value
HIV overall						
Year *t*	0.06 (0.01)	< 0.001	0.08 (0.03)	0.019	0.03 (0.04)	0.421
Vasectomy rate *v*	-	-	-0.007 (0.01)	0.510	0.08 (0.05)	0.139
Interaction *t* x *v*	-	-	-	-	-0.01 (0.003)	0.100
β_0_/AIC	-8.69/144.1		-9.21/145.7		-9.58/145.26	
HIV in men						
Year *t*	0.06 (0.01)	< 0.001	0.09 (0.04)	0.019	0.04 (0.04)	0.418
Vasectomy rate *v*	-	-	-0.01 (0.01)	0.519	0.09 (0.06)	0.133
Interaction *t* x *v*	-	-	-	-	-0.01 (0.004)	0.095
β_0_/AIC	-8.69/142.04		-8.7/143.63		-9.12/143.16	
HIV in women						
Year *t*	0.03 (0.01)	< 0.001	0.05 (0.03)	0.055	0.02 (0.03)	0.598
Vasectomy rate *v*	-	-	-0.01 (0.01)	0.475	0.05 (0.04)	0.224
Interaction *t* x *v*	-	-	-	-	-0.004 (0.003)	0.169
β_0_/AIC	-10.22/102.17		-10.23/103.67		-10.47/103.94	
Syphilis						
Year *t*	0.04 (0.01)	< 0.001	0.10 (0.03)	0.726	-0.01 (0.04)	0.833
Vasectomy rate *v*	-	-	0.01 (0.01)	0.232	0.04 (0.05)	0.396
Interaction *t* x *v*	-	-	-	-	-0.002 (0.03)	0.520
β_0_/AIC	-8.59/151.15		-8.58/151.8		-8.71/153.4	
Gonorrhea						
Year *t*	0.08 (0.01)	< 0.001	0.02 (0.03)	0.390	0.05 (0.03)	0.115
Vasectomy rate *v*	-	-	0.021 (0.01)	0.008	-0.03 (0.04)	0.440
Interaction *t* x *v*	-	-	-	-	0.004 (0.003)	0.190
β_0_/AIC	-9.8/132.82		-9.78/129.6		-9.55/130	
Hepatitis B						
Year *t*	0.08 (0.04)	0.037	0.30 (0.08)	< 0.001	0.27 (0.11)	0.017
Vasectomy rate *v*	-	-	-0.08 (0.03)	0.002	-0.02 (0.15)	0.809
Interaction *t* x *v*	-	-	-	-	-0.004 (0.01)	0.690
β_0_/AIC	-10.3/146.9		-10.35/142.57		-10.59/144.4	

AIC: Akaike information criterion; β_0_: intercept of the model; SE: standard error of the coefficient.

Note: *t*: 2008, ..., 2017.

Table 4 displays the results of the interaction term between time *t* and vasectomy rate *v* in Model 3 stratified according to age group. The slope of the interaction term in the 50-59 year age group showed a positive effect on the incidence rates of gonorrhea (0.015, p = 0.002). While the vasectomy rates increased during the study period, the incidence rate of gonorrhea also increased. By contrast, the changes in vasectomy rates were inversely associated with HIV (-0.01, p = 0.002) in the 40-49 year age group and the incidence rates of hepatitis B in the 20-29 year age group (-0.075, p = 0.003).

**Table 4:** Negative binomial for sexually transmitted diseases (STD) trends in Chile from 2008 to 2017, and its relationship with vasectomy rates stratified by age groups.

	Age groups (years)							
	20-29		30-39		40-49		50-59	
	Coefficient (SE)	p-value	Coefficient (SE)	p-value	Coefficient (SE)	p-value	Coefficient (SE)	p-value
HIV								
Year *t*	0.07 (0.02)	< 0.001	0.05 (0.04)	0.293	-0.02 (0.04)	0.700	0.10 (0.03)	0.002
Vasectomy rate *v*	0.12 (0.13)	0.326	0.032 (0.03)	0.282	0.08 (0.03)	0.011	-0.02 (0.09)	0.835
Interaction *t* x *v*	-0.012 (0.01)	0.266	-0.003 (0.002)	0.207	-0.01 (0.002)	0.002	-0.09 (0.09)	0.816
β_0_/AIC	-9.34/146.9		-9.44/146.183		-9.7/142.3		-9.2/146.6	
Syphilis								
Year *t*	0.02 (0.015)	0.277	-0.01 (0.04)	0.722	-0.03 (0.05)	0.455	0.04 (0.03)	0.156
Vasectomy rate *v*	0.02 (0.09)	0.866	0.03 (0.03)	0.313	0.04 (0.03)	0.182	-0.10 (0.08)	0.165
Interaction *t* x *v*	0.004 (0.01)	0.680	-0.001 (0.002)	0.452	-0.002 (0.002)	0.249	0.01 (0.24)	0.167
β_0_/AIC	-8.55/151.7		-8.7/153		-8.8/152.5		-8.3/153.3	
Gonorrhea								
Year *t*	0.05 (0.01)	< 0.001	0.05 (0.03)	0.116	0.07 (0.04)	0.078	0.03 (0.02)	0.140
Vasectomy rate *v*	-0.03 (0.08)	0.728	-0.016 (0.03)	0.472	-0.03 (0.03)	0.277	-0.14 (0.06)	0.017
Interaction *t* x *v*	0.01 (0.01)	0.198	0.002 (0.002)	0.189	0.003 (0.003)	0.122	0.015 (0.01)	0.002
β_0_/AIC	-9.7/129.1		-8.7/151.5		-9.5/129.6		-9.3/128.9	
Hepatitis B								
Year *t*	0.17 (0.04)	< 0.001	0.22 (0.11)	0.051	0.36 (0.12)	0.004	0.33 (0.07)	< 0.001
Vasectomy rate *v*	0.69 (0.29)	0.016	0.03 (0.08)	0.724	-0.08 (0.08)	0.339	-0.05 (0.18)	0.795
Interaction *t* x *v*	-0.075 (0.03)	0.003	-0.005 (0.01)	0.355	0.002 (0.01)	0.668	-0.02 (0.01)	0.256
β_0_/AIC	-11.1/143.9		-10.8/144.5		10.2/143.4		-10.6/141.6	

AIC: Akaike information criterion; β_0_: intercept of the model; SE: standard error of the coefficient.

Note: *t*: 2008, ..., 2017.

## Discussion

Our results indicate a significant annual increase in vasectomy rates during the last 10 years in Chile, with substantial differences between the age groups. Our findings differ from those reported in the United States, where the trend was downward during a similar period ^3^ ^,^ ^11^. Men aged 30-49 years accounted for the highest increase in vasectomy rates in this study, as expected, and similar to another report ^13^. On the contrary, the vasectomy trends in the youngest age group (20-29 years) were stationary but increased during the last studied year. Comparisons with evidence are difficult since studies are constantly emerging, exogenous factors differ, and existing studies have only cross-sectionally described vasectomy rates in nonrepresentative samples ^4^. Nevertheless, vasectomy and other contraceptive methods should be stand out for surveillance, and whether the use of definitive contraceptive methods continue to increase in men without offspring and in the youngest groups should be investigated, since this could impact the demographic structure of the population, considering that Chile already has a low fertility rate ^14^. Moreover, further research regarding men’s motivation for elective vasectomy, related risky sexual behaviors, and later vasectomy reversal is necessary.

Numerous exogenous and endogenous factors could explain the increase in vasectomy rates in the study population ^13^. Some examples are the improved public awareness and education about vasectomy as a safe and effective method; cultural shifts toward family planning and gender roles that impact the acceptability of vasectomy; economic factors related to the cost of raising children and the desire for smaller families; and promotion campaigns and advancements in medical technology availability. However, our findings show that most vasectomies were performed in Chilean private healthcare system, which suggests that access and funding contribute to inequalities in sexual and reproductive healthcare rights. From 2021 onward, the Chilean public healthcare system allows beneficiaries to access private providers of elective vasectomy at an affordable price. This new scenario could encourage more men to undergo vasectomy, which will require further public health surveillance and impact evaluation.

From a population-based perspective, vasectomy rates were cross-sectionally and positively associated with the incidence of gonorrhea and inversely associated with the incidence of hepatitis B in the overall study population, but not with the incidence of HIV or syphilis. This study analysis was probably less sensitive to detecting the association with the incidence of HIV, given the long disease course of HIV/AIDS and the diagnosis and notification delay in the surveillance system in Chile. Similarly, the acute syphilis phase is characterized by a painless lesion that can go unnoticed. Syphilis also requires a second test to confirm the diagnosis. On the other hand, since asymptomatic gonorrheal infection in men is rare and laboratory notification is mandatory and immediate in Chile, gonorrhea would be a reliable surrogate indicator of any STD. Regarding hepatitis B infection, 70% of patients are asymptomatic, and the effect of mass vaccination incorporated into the Chilean immunization program for the infant population in 2015 may explain the inverse association.

All STD cases analyzed in this study increased from 2008 to 2017, despite WHO reporting a 31% reduction in global HIV cases from 2010 to 2020, including the Americas ^(^ ^15^. The correlation patterns used to estimate whether vasectomy explains these STD increments differed according to age groups. In the 20-29 years group, the increment in the incidence rate of hepatitis B over the years was attenuated by higher vasectomy rates. Similar results were found for HIV in the 40-49 years age group. On the other hand, the increment in the incidence rate of gonorrhea in the 50-59 years age group was exacerbated by higher vasectomy rates. These findings suggest that self-care behaviors or sexual education related to a voluntary vasectomy choice differ by age and sex. For example, in 2010, the Sexual Education Law was promulgated in Chile, which stipulated that educational establishments must implement a sexual education program concerning affectivity, sexuality, and gender from a human rights perspective at the primary and secondary education levels. Hence, vasectomy choice in younger age groups may be related to a greater awareness of fertility regulation and self-care measures, contrary to that in older ages with risky sexual behaviors.

In summary, the relationship between vasectomy and STD is not direct, since vasectomy is a surgical procedure for male sterilization and is not biologically linked to an increased risk of STD. However, besides behavioral factors, other contextual factors might explain this indirect relationship. For instance, healthcare-seeking behavior since individuals who undergo vasectomy are typically more proactive about their reproductive health and may be more likely to seek regular testing for STD. Public health campaigns and educational materials may also inadvertently influence perceptions about vasectomy and STD. Confounding variables, such as age, socioeconomic status, education level, may influence both the decision to undergo vasectomy and the risk of STD. Finally, if data collection were not random or valid, it could skew or bias the observed associations.

To our knowledge, this is the first study to report a 10-year vasectomy trends using representative population-based data in a Latin America country. In Chile, notifications for hospital discharges and their causes are mandatory in public and private healthcare system. Since vasectomy is a hospital procedure, the database used in this study is representative of the population. The main limitations of this study are related to the design. Since this is an ecological study, no individual-level conclusions can be drawn from our results, and only non-causal inferences can be made from the observed relationships. However, this study used the most efficient study design to formulate new hypotheses for future research. Finally, our data do not necessarily reflect the time of infection, especially for HIV and hepatitis B, and the surveillance systems could potentially present underreporting.

In conclusion, vasectomy trend rates significantly increased from 2018 to 2017 in Chile, especially among men aged 30-49 years, as well as STD, highlighting the importance of surveillance and planning new prevention strategies. High vasectomy rates could impact the STD trends according to age. However, studies at the individual level are necessary to characterize this population and evaluate if vasectomy is a new risk factor for targeting preventive intervention and reproductive sexual and health policies to aid in controlling STD epidemics.
